# Supplementary material for: Chemical Composition of Various Nepeta cataria Plant Organs’ Methanol Extracts Associated with In Vivo Hepatoprotective and Antigenotoxic Features as well as Molecular Modeling Investigations
Source: Plants (Basel). 2022 Aug 14;11(16):2114. doi: 10.3390/plants11162114 (PMC9415533; doi:10.3390/plants11162114)
Supplement: Supplementary file 1 [file plants-11-02114-s001.zip › plants-1737280-supplementary.pdf]

Supplementary Materials

for

Chemical composition of various *Nepeta cataria* plant organs' methanol extracts associated with the *in vivo* hepatoprotective and antigenotoxic features, and molecular modeling investigations

Milena D. Vukić [1], Nenad Vuković [1,*], Milan Mladenović [1], Nevena Tomašević [1], Sanja Matić [3], Snezana Stanić [4], Filippo Sapienza[5], Rino Ragno [5], Mijat Božović [6], Miroslava Kacaniova [7,8]

[1] Department of Chemistry, Faculty of Science, University of Kragujevac, Radoja Domanovića 12, 34000 Kragujevac, Serbia
[2] Kragujevac Center for Computational Biochemistry, Department of Chemistry, Faculty of Science, University of Kragujevac, Radoja Domanovića 12, 34000 Kragujevac, Serbia
[3] Department of Science, Institute for Information Technologies Kragujevac, University of Kragujevac, Jovana Cvijića bb, 34000 Kragujevac, Serbia
[4] Department of Biology and Ecology, Faculty of Science, University of Kragujevac, Radoja Domanovića 12, 34000 Kragujevac, Serbia
[5] Rome Center for Molecular Design, Department of Drug Chemistry and Technology, Faculty of Pharmacy and Medicine, Sapienza University of Rome, Piazzale Aldo Moro 5, 00185 Rome, Italy
[6] Faculty of Science and Mathematics, University of Montenegro, Džordža Vašingtona bb, 81000 Podgorica, Montenegro
[7] Institute of Horticulture, Faculty of Horticulture and Landscape Engineering, Slovak University of Agriculture in Nitra, Tr. A. Hlinku 2, 94976 Nitra, Slovakia
[8] Department of Bioenergy, Food Technology and Microbiology, Institute of Food Technology and Nutrition, University of Rzeszow, 4 Zelwerowicza St., 35601 Rzeszow, Poland
*Correspondence: nenad.vukovic@pmf.kg.ac.rs; Tel.: +381336223

Content

Figure S1. The bioactive conformations of protocatechuic acid (A), *p*-hydroxybenzoic acid (B), caffeic acid (C), and syringic acid (D) within the *r*CATCmpd I. Amino acid residues are depicted in white, hem containing the oxoferryl porphyrin π-cation radical is colored green. For the clarity of presentation, only polar hydrogen atoms were presented within the naturally occurring compounds.

Figure S2. The bioactive conformations of *p*-coumaric acid (A), ferulic acid (B), luteolin-7-*O*-glucoside (C), and quercetin-3-*O*-glucoside (D) within the *r*CATCmpd I. Amino acid residues are depicted in white, hem containing the oxoferryl porphyrin π-cation radical is colored green. For the clarity of presentation, only polar hydrogen atoms were presented within the naturally occurring compounds.

Figure S3. The bioactive conformations of quercetin-3-*O*-rutinoside (A), kaempferol-3-*O*-glucoside (B), quercitrin (C), luteolin (D), kaempferol (E), and isorhamnetin (F) within the *r*CATCmpd I. Amino acid residues are depicted in white, hem containing the oxoferryl porphyrin π-cation radical is colored green. For the clarity of presentation, only polar hydrogen atoms were presented within the naturally occurring compounds.

Figure S4. The bioactive conformations of protocatechuic acid (A), *p*-hydroxybenzoic acid (B), caffeic acid (C), and syringic acid (D) within the *r*DNA binding and cleavage domain of *r*TopII*α*. All of the obtained bioactive conformations were superimposed to MDA illustrated in yellow. The sense strand was depicted in green, the antisense strand was colored purple. The nucleotides of interest are depicted in white, whereas the remaining ones were displayed by blue nucleotides objects. For the clarity of presentation, the *C*-terminal domain of *r*TopII*α*, nesting the targeted compounds, was illustrated by pink sphere, whereas the non-targeted *C*-terminal domain was denoted by pink ribbons.

Figure S5. The bioactive conformations of *p*-coumaric acid (A), ferulic acid (B), luteolin-7-*O*-glucoside (C), and quercetin-3-*O*-glucoside (D) within the *r*DNA binding and cleavage domain of *r*TopII*α*. All of the obtained bioactive conformations were superimposed to MDA illustrated in yellow. The sense strand was depicted in green, the antisense strand was colored purple. The nucleotides of interest are depicted in white, whereas the remaining ones were displayed by blue nucleotides objects. For the clarity of presentation, the *C*-terminal domain of *r*TopII*α*, nesting the targeted compounds, was illustrated by pink sphere, whereas the non-targeted *C*-terminal domain was denoted by pink ribbons.

Figure S6. The bioactive conformations of quercetin-3-*O*-rutinoside (A), kaempferol-3-*O*-glucoside (B), quercitrin (C), luteolin (D), kaempferol (E), and isorhamnetin (F) within the *r*DNA binding and cleavage domain of *r*TopII*α*. All of the obtained bioactive conformations were superimposed to MDA illustrated in yellow. The sense strand was depicted in green, the antisense strand was colored purple. The nucleotides of interest are depicted in white, whereas the remaining ones were displayed by blue nucleotides objects. For the clarity of presentation, the *C*-terminal domain of *r*TopII*α*, nesting the targeted compounds, was illustrated by pink sphere, whereas the non-targeted *C*-terminal domain was denoted by pink ribbons.

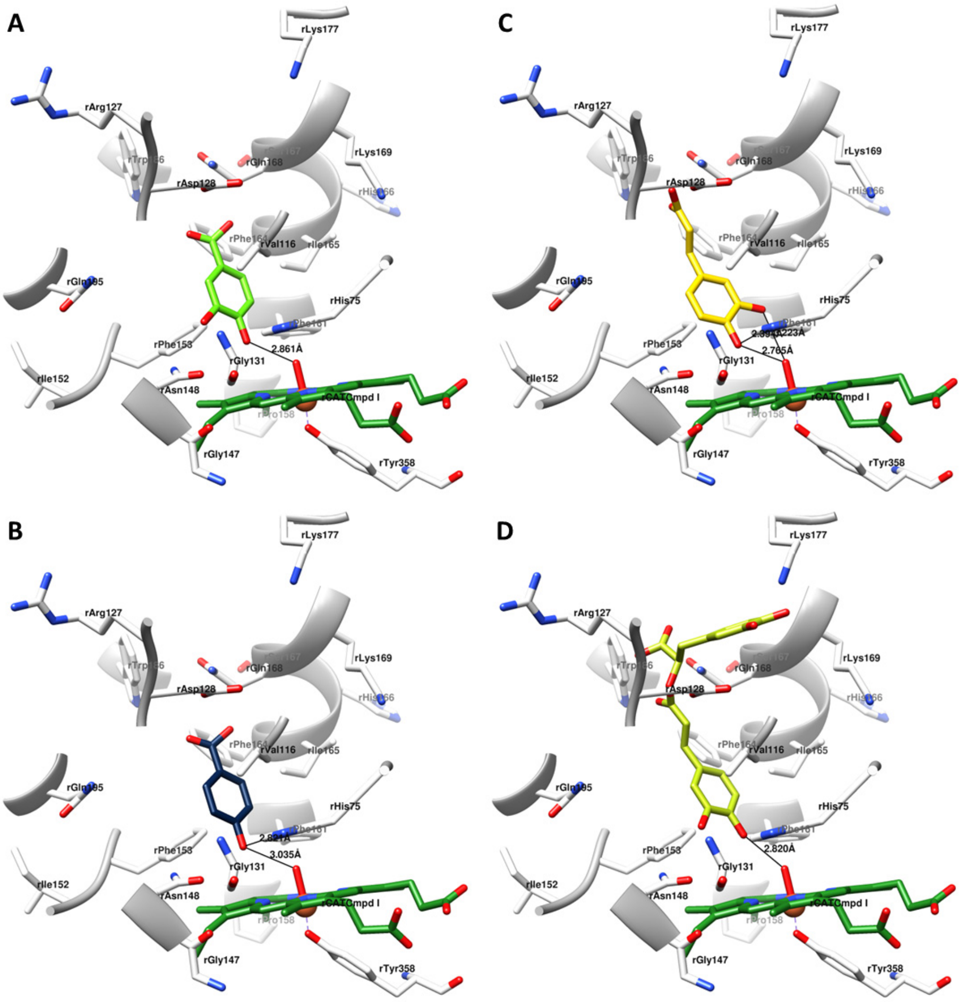

Figure S1. The bioactive conformations of protocatechuic acid (A), *p*-hydroxybenzoic acid (B), caffeic acid (C), and syringic acid (D) within the *r*CATCmpd I. Amino acid residues are depicted in white, hem containing the oxoferryl porphyrin π-cation radical is colored green. For the clarity of presentation, only polar hydrogen atoms were presented within the naturally occurring compounds.

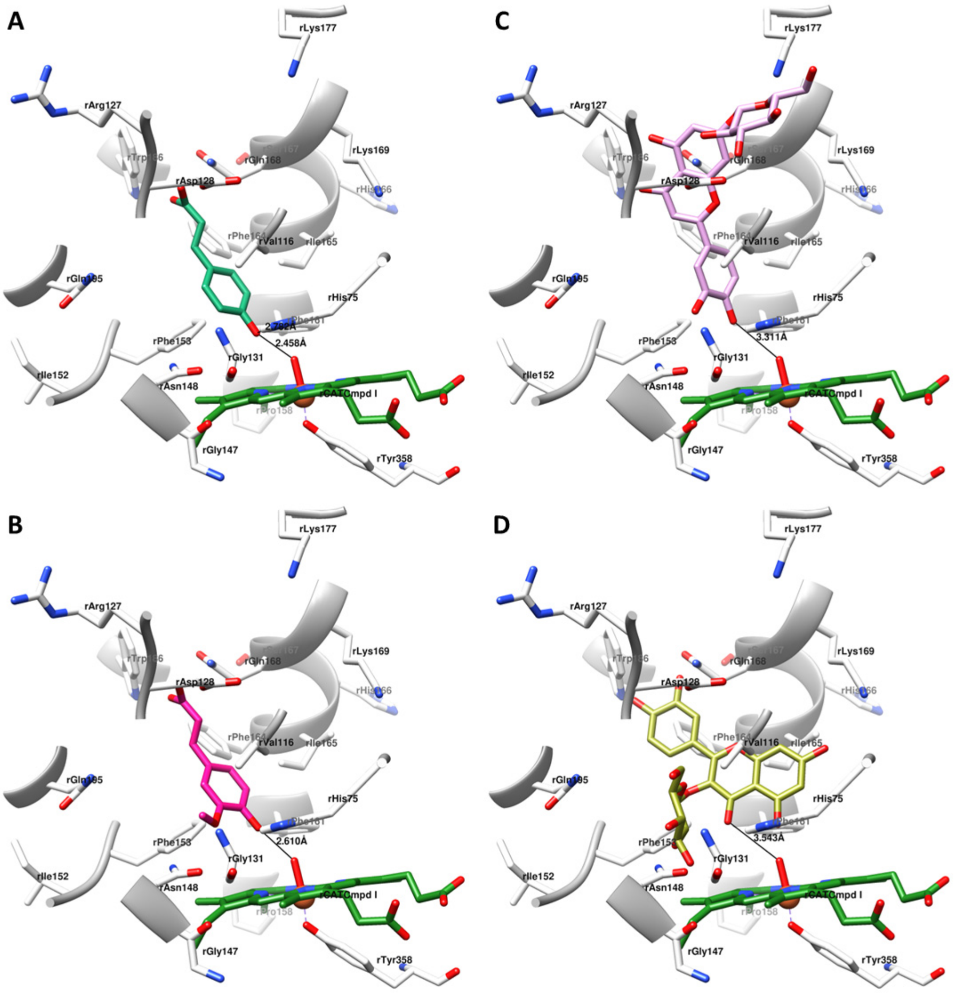

Figure S2. The bioactive conformations of *p*-coumaric acid (A), ferulic acid (B), luteolin-7-*O*-glucoside (C), and quercetin-3-*O*-glucoside (D) within the *r*CATCmpd I. Amino acid residues are depicted in white, hem containing the oxoferryl porphyrin π-cation radical is colored green. For the clarity of presentation, only polar hydrogen atoms were presented within the naturally occurring compounds.

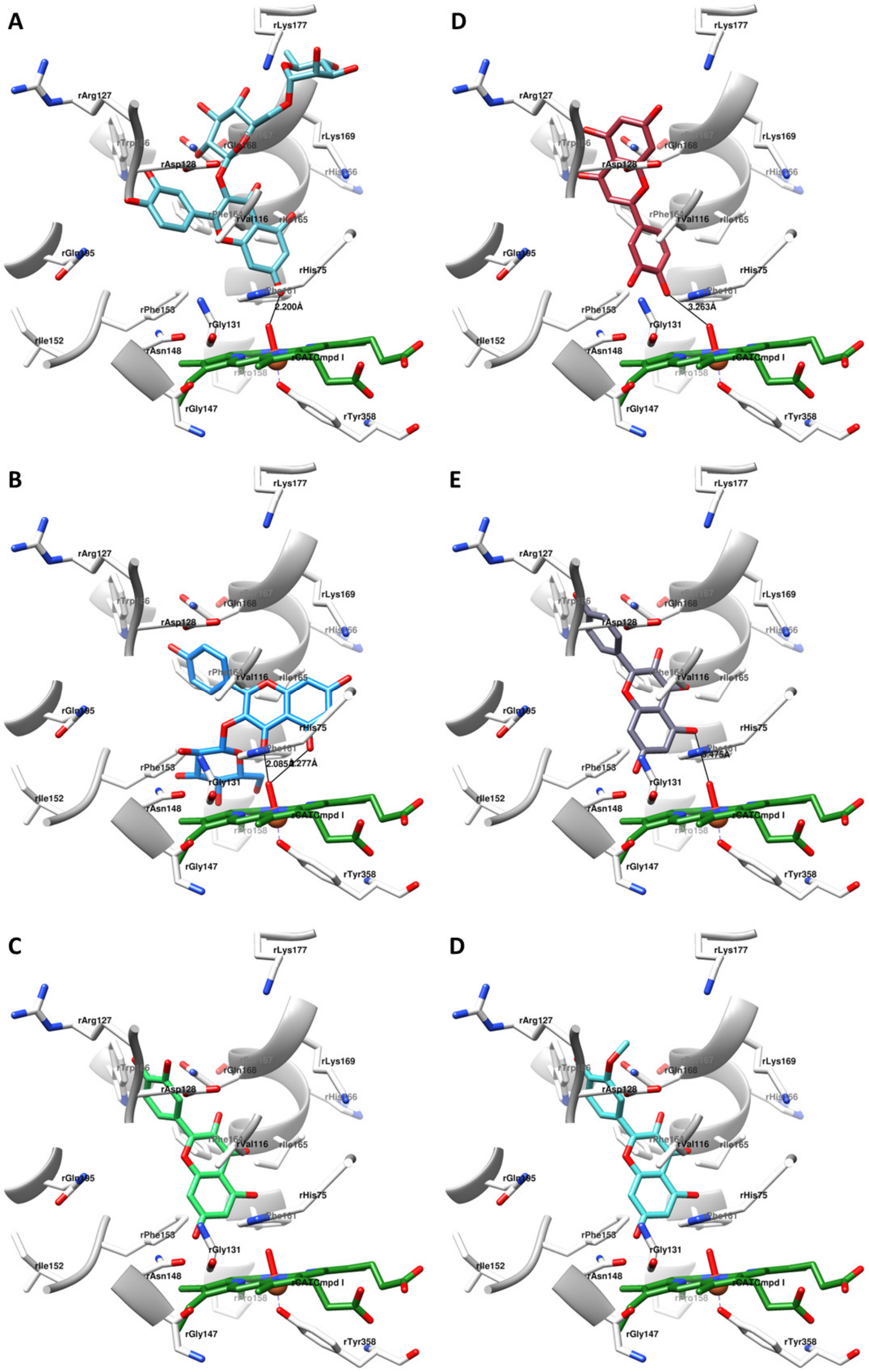

Figure S3. The bioactive conformations of quercetin-3-*O*-rutinoside (A), kaempferol-3-*O*-glucoside (B), quercitrin (C), luteolin (D), kaempferol (E), and isorhamnetin (F) within the *r*CATCmpd I. Amino acid residues are depicted in white, hem containing the oxoferryl porphyrin π-cation radical is colored green. For the clarity of presentation, only polar hydrogen atoms were presented within the naturally occurring compounds.

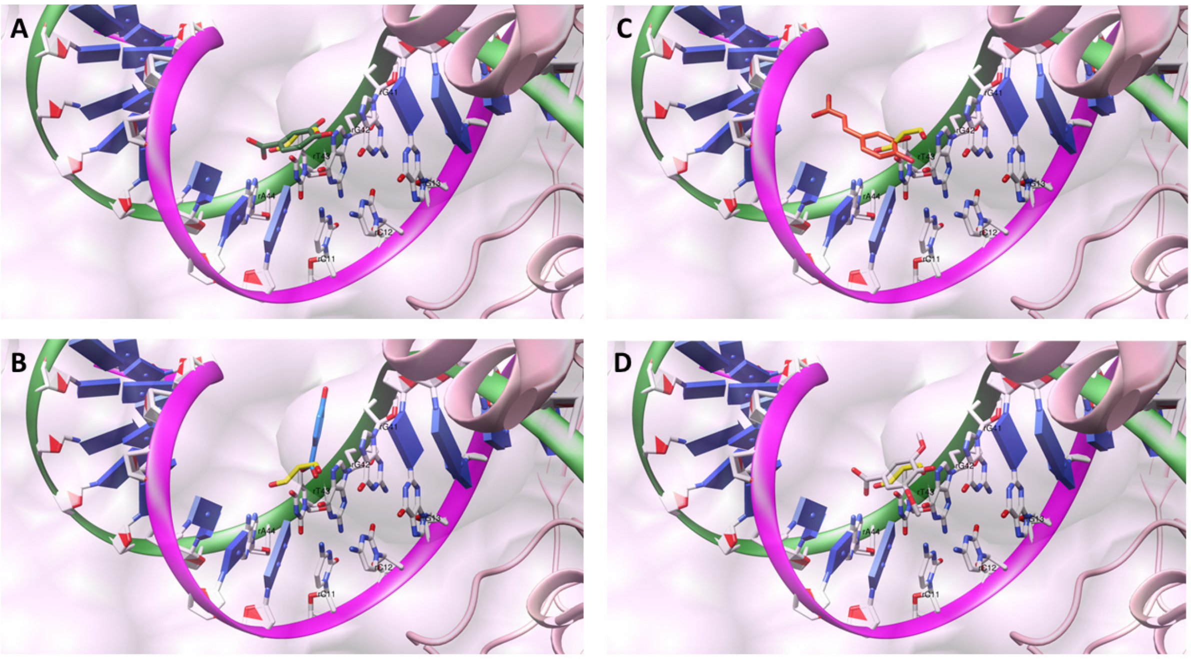

Figure S4. The bioactive conformations of protocatechuic acid (A), *p*-hydroxybenzoic acid (B), caffeic acid (C), and syringic acid (D) within the *r*DNA binding and cleavage domain of *r*TopII*α*. All of the obtained bioactive conformations were superimposed to MDA illustrated in yellow. The sense strand was depicted in green, the antisense strand was colored purple. The nucleotides of interest are depicted in white, whereas the remaining ones were displayed by blue nucleotides objects. For the clarity of presentation, the *C*-terminal domain of *r*TopII*α*, nesting the targeted compounds, was illustrated by pink sphere, whereas the non-targeted *C*-terminal domain was denoted by pink ribbons.

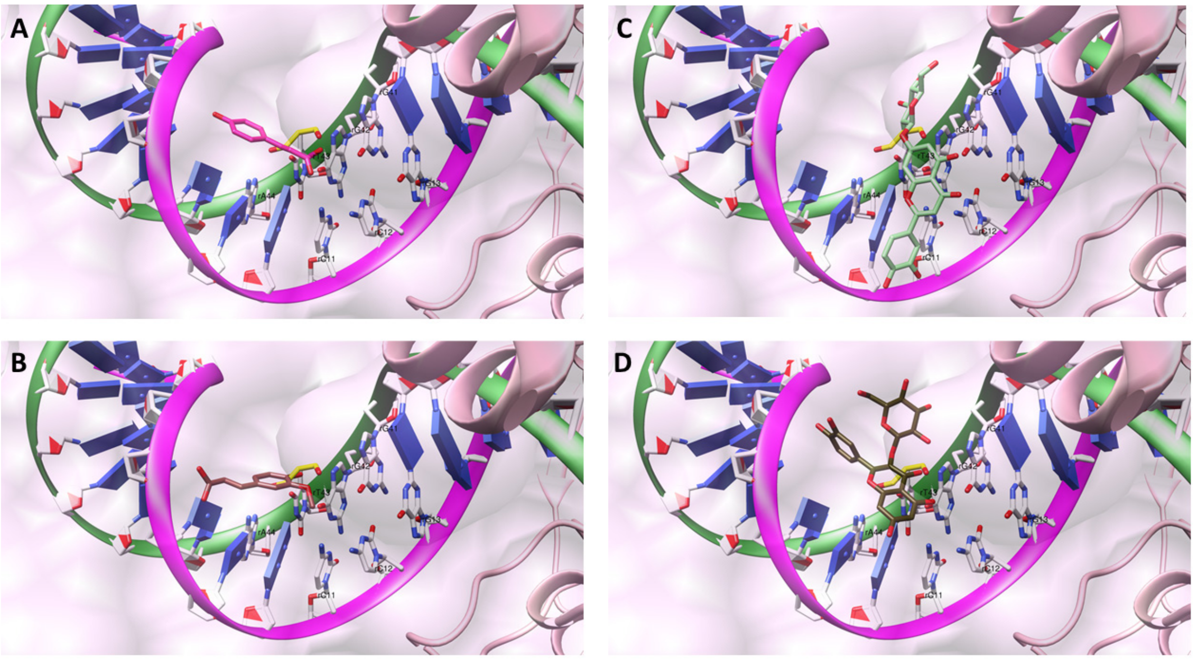

Figure S5. The bioactive conformations of *p*-coumaric acid (A), ferulic acid (B), luteolin-7-*O*-glucoside (C), and quercetin-3-*O*-glucoside (D) within the *r*DNA binding and cleavage domain of *r*TopIIα. All of the obtained bioactive conformations were superimposed to MDA illustrated in yellow. The sense strand was depicted in green, the antisense strand was colored purple. The nucleotides of interest are depicted in white, whereas the remaining ones were displayed by blue nucleotides objects. For the clarity of presentation, the *C*-terminal domain of *r*TopIIα, nesting the targeted compounds, was illustrated by pink sphere, whereas the non-targeted *C*-terminal domain was denoted by pink ribbons.

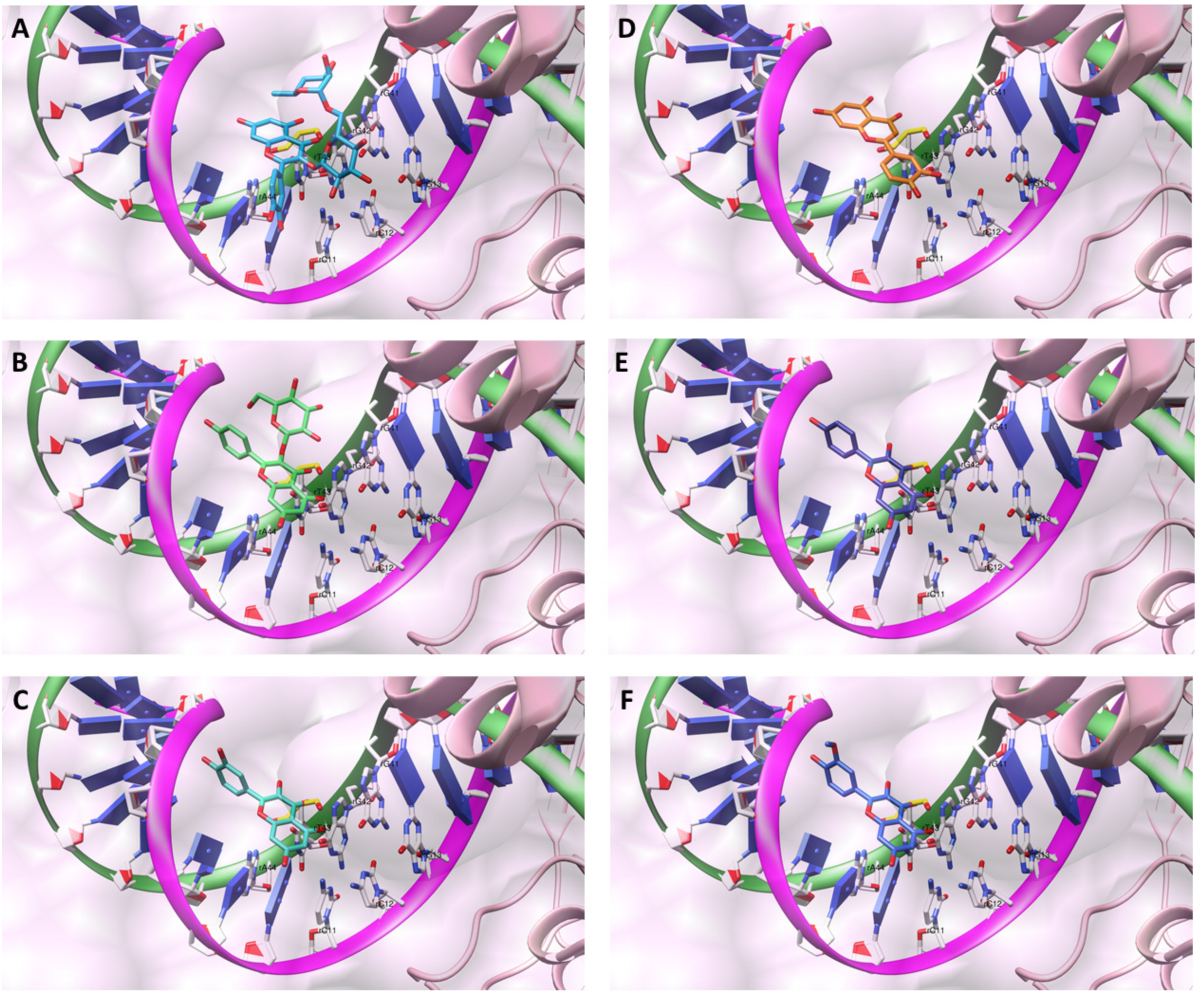

Figure S6. The bioactive conformations of quercetin-3-*O*-rutinoside (A), kaempferol-3-*O*-glucoside (B), quercitrin (C), luteolin (D), kaempferol (E), and isorhamnetin (F) within the *r*DNA binding and cleavage domain of *r*TopIIα. All of the obtained bioactive conformations were superimposed to MDA illustrated in yellow. The sense strand was depicted in green, the antisense strand was colored purple. The nucleotides of interest are depicted in white, whereas the remaining ones were displayed by blue nucleotides objects. For the clarity of presentation, the *C*-terminal domain of *r*TopIIα, nesting the targeted compounds, was illustrated by pink sphere, whereas the non-targeted *C*-terminal domain was denoted by pink ribbons.
